# Effects of four non-invasive stimulations on swallowing function and quality of life of stroke patients—a network meta-analysis

**DOI:** 10.3389/fnhum.2025.1519660

**Published:** 2025-03-13

**Authors:** Xinyu Lin, Haojie Li, Xie Wu, Rui Huang

**Affiliations:** School of Exercise and Health, Shanghai University of Sport, Shanghai, China

**Keywords:** stroke, noninvasive electrical stimulation, swallowing function, quality of life, rehabilitative

## Abstract

**Background:**

Stroke is a sudden neurological disorder that causes severe neurological damage mainly due to lack of oxygen to brain cells as a result of interruption of blood flow to the brain. Dysphagia is a common problem in stroke patients, interfering with diet and nutrition and possibly leading to complications. About 50–80% of stroke patients experience dysphagia in the acute phase, which may lead to serious consequences such as aspiration and pneumonia. Therefore, improving swallowing function is essential to enhance patients’ quality of life (QoL). Traditional rehab methods are limited, but non-invasive stimulation is safer and improves swallowing function through various mechanisms: pharyngeal electrical stimulation (PES) boosts cortical excitability and plasticity by stimulating pharyngeal nerves; neuro-muscular electrical stimulation (NmeS) enhances infrahyoid muscle strength and mobility with low-frequency pulses; repetitive transcranial magnetic stimulation (rTMS) promotes motor cortex remodeling; transcranial direct current stimulation (tDCS) increases neural activity in swallowing-related regions. These techniques are safe, easy to use, and show great potential for clinical application, needing further study.

**Methods:**

Six databases were systematically searched, and 17 randomized controlled trials with 788 stroke patients were finally included. The outcome indicators were swallowing function and QoL related indicators. Net meta-analysis was performed using Stata 17.0 to assess the relative effectiveness of each combined intervention and to test the consistency of direct and indirect evidence.

**Results:**

For swallowing function, rTMS [SMD = 5.10, 95% CI (3.20, 7.01), *p* < 0.0001, SUCRA = 87.3] showed the best results. For QoL, NmeS [SMD = 3.51, 95% CI (0.54, 6.47), *p* < 0.0001, SUCRA = 79.3] shows all its unique advantages.

**Conclusion:**

rTMS can effectively improve the swallowing function of stroke patients, while NmeS has the best effect in improving the QoL.

**Systematic review registration:**

https://www.crd.york.ac.uk/PROSPERO/view/CRD42024603146

## 1 Introduction

Stroke is a sudden onset neurological disease that is mainly due to the interruption of cerebral blood flow supply, resulting in hypoxia and nutritional deficiencies in brain cells, which in turn causes damage to neurological function. According to the World Health Organization, stroke is an important cause of death and disability worldwide, with a higher prevalence especially in the elderly population (Ingall et al., 2000). Stroke not only affects patients’ motor, cognitive, and speech functions, but also severely impairs swallowing function. Dysphagia is a common problem in stroke patients in the acute phase, which may lead to the inability of patients to swallow by themselves in severe cases and become an important indicator in their rehabilitation process (Martino et al., 2005). During the rehabilitation process, impaired swallowing function not only affects the patient’s diet and nutritional intake, but may also lead to complications such as dehydration and malnutrition, thus prolonging the rehabilitation cycle and increasing medical costs (Nilsson et al., 1998). Therefore, studying the impairment of swallowing function and its recovery strategies in stroke patients is crucial to improve their quality of life (QoL).

The incidence of dysphagia is higher in stroke patients, particularly during the acute phase, with approximately 50–80% of stroke patients experiencing varying degrees of dysphagia (Yang et al., 2012). While some patients may see an improvement in swallowing function as they recover from the acute phase, others may continue to face dysphagia throughout the recovery phase. Hypophagia may lead to serious consequences such as aspiration, bronchospasm, airway obstruction and asphyxia, as well as increase the risk of pneumonia (Banda et al., 2022). Misaspiration is one of the common complications after stroke, which may directly lead to death or significantly reduce the QoL (Labeit et al., 2023). Therefore, improving swallowing function not only helps to minimize complications, but also improves the overall QoL of patients. Studies have shown that the QoL of stroke patients is significantly improved after rehabilitation, which is closely related to the recovery of their swallowing function (Tarihci Cakmak et al., 2023; Gao and Zhang, 2017). The QoL of patients should not be neglected during a long period of rehabilitation, so it is necessary to combine the recovery of swallowing function to improve the overall QoL of patients.

For the rehabilitation of swallowing function, commonly used methods include swallowing training, dietary modification, and speech therapy. These methods usually improve swallowing function by improving patients’ swallowing skills, adjusting dietary structure, and providing psychological support (Bath et al., 2018). For example, certain studies have shown that regular swallowing training can effectively enhance patients’ swallowing ability, thereby reducing the risk of aspiration (Wilkinson et al., 2021). However, these traditional methods mainly focus on improving swallowing function rather than directly intervening in the brain and nervous system. Therefore, there is an urgent need to explore novel therapeutic modalities for post-stroke swallowing dysfunction in order to improve therapeutic efficacy and patients’ QoL.

Non-invasive stimulation is an emerging treatment developed in recent years, which mainly includes neuromuscular electrical stimulation (NmeS), pharyngeal electrical stimulation (PES), repetitive transcranial magnetic stimulation (rTMS) and transcranial direct current stimulation (tDCS). These methods have demonstrated good potential for clinical application in improving neurological function in stroke patients (Shen et al., 2022). PES enhances swallowing function by stimulating the sensory nerves of the pharyngeal mucosa, increasing cortical motor excitability for swallowing, and promoting neural plasticity. NmeS improves swallowing function by using low-frequency pulse electrical stimulation to target the infrahyoid muscle group, enhancing muscle contraction strength and increasing the mobility of structures involved in swallowing. It has been shown that rTMS can promote neural remodeling in the motor cortex, thereby improving motor function (Liao et al., 2024). tDCS has also been shown to enhance neural activity in swallowing-related brain regions and improve swallowing ability (Hurley and Machado, 2017). The advantages of these non-invasive stimulation methods are that they are safe, easy to operate, and do not require surgical or pharmacological intervention, which can promote the recovery of neurological function more effectively. However, these methods also have certain limitations, such as the effect varies according to individual differences, as well as the possibility of discomfort in some patients (Blumberger et al., 2018). At present, research on the application of non-invasive stimulation in stroke patients is still relatively scarce, so this paper will select four commonly used non-invasive stimulation methods to be discussed, aiming to provide scientific basis and innovative ideas for clinical treatment.

In summary, stroke is a disease that seriously affects the QoL of patients, and swallowing dysfunction is one of its common complications, which urgently requires the exploration of effective rehabilitation methods. Current traditional swallowing function rehabilitation methods have limitations in improving swallowing ability, so the introduction of non-invasive stimulation methods is of great clinical significance. This study will focus on analyzing the effects of four non-invasive stimuli on swallowing function and QoL of stroke patients, and is expected to provide new insights and directions for research and clinical practice in related fields.

## 2 Methods

This study was guided by the Preferred Reporting Items for Systematic Evaluation and Meta-Analysis (PRISMA list for NMAs10 and the Cochrane Handbook for the Evaluation of Intervention Systems). Registration number: CRD42024603146.

### 2.1 Data sources

In this study, the process of searching, inclusion, screening, and exclusion criteria of the literature were strictly followed as stipulated in the PRIMSA entries and in accordance with the PICOS principles of evidence-based medicine. Systematic searches were performed on PubMed, Embase, Web of Science, Cochrane, EBSCO, and China National Knowledge Infrastructure (CNKI), and the selection of included studies was done independently by two researchers (HL and XL). Searches were performed in PubMed and Cochrane using terms in MeSH. Searches were performed in Embase using terms in Emtree, and in CNKI using subject terms combined with free terms. The reference lists of relevant articles were also manually screened for other studies that might be eligible. The search timeframe was from January 1980 ending October 2024. This study only included human trials published in English or Chinese, with Chinese articles sourced exclusively from Chinese core journals. These core journals include the Chinese Social Sciences Citation Index (CSSCI) by Nanjing University, the Peking University Core Journal List by Peking University, the Chinese Science and Technology Core Journals by the Institute of Scientific and Technical Information of China, and the core journals indexed in the Chinese Science Citation Database (CSCD).

The search strategy followed the PICOS principle of evidence-based medicine: (P) Population: stroke patients; (I) Interventions: NmeS, PES, rTMS, tDCS; (C) Controls: routine swallowing rehabilitation or no intervention; (O) Outcomes: indicators reflecting swallowing function, e.g., Functional Dysphagia Scale (FDS), Functional Oral Intake Scale (FoiS), video fluoroscopic swallowing study (VFSS), Dysphagia Severity Rating Scale (DSRS), Penetration Aspiration Scale (PAS), and Dysphagia Outcome and Severity Scale (DOSS), and indicators reflecting QoL, e.g., Barthel index (BI), Quality of Life Assessment the Swallowing Quality of Life (SWAL-QOL), American Speech-Language-Hearing Association National Outcome Measurement System (ASHA NOMS), Clinical Dysphagia Scale (CDS) and Modified Mann Assessment of Swallowing Ability (MASA); (S) Study type: RCTs.

### 2.2 Study selection

To (“stroke” or “cerebral hemorrhage” or “stroke” or “cerebral infarction” or “cerebral embolism”) and (“transcranial magnetic” or “electrostimulation” or “transcranial direct current stimulation”) (“cerebral embolism”) and (“transcranial magnetic” or “electrical stimulation” or “transcranial direct current stimulation” or “neuromuscular electrical stimulation” or “neuromuscular electrical stimulation” or “neuromuscular electrical stimulation” or “neuromuscular electrical stimulation”). (“Neuromuscular electrical stimulation” or “pharyngeal electrical stimulation”) and (“swallowing” or “quality of life” or “ability to perform activities of daily living”) and (“randomized controlled trial” or “controlled clinical trial”) were searched in Chinese; the search was based on (“stroke”) and (“Electrical Stimulation” or “Translational Stimulation”). (“Electrical Stimulation” or “Transcranial Magnetic Stimulation” or “Transcranial Direct Current Stimulation” or “Deep Brain Stimulation”) and (“Deglutition” or “Degree”). (“Deglutition” or “Deglutition Disorders” or “Quality of Life”) and (“Randomized controlled trial” or “RCT”) and (“Randomized controlled trial” or “Controlled clinical trial” or “Randomized”) were searched for English subject terms.

Reference lists of relevant articles were manually screened for potentially eligible studies. The obtained literature was screened. Duplicate entries were first eliminated by endnote automatic weight checking, and then manually removed by reading the headings for duplicate literature searches. The remaining literature was further screened to eliminate non-stroke disease studies, studies that did not assess swallowing function or ability to perform activities of daily living, studies without repetitive transcranial magnetic stimulation, studies that were not in the combined category, reviews, conference abstracts, animal studies, research protocols, and book chapters.

### 2.3 Eligibility criteria

We included randomized clinical trials in people with confirmed acute or chronic stroke (RCTs) that compared the effects of different noninvasive stimuli on swallowing function and QoL in stroke patients.

Studies were eligible for inclusion if they met the following criteria: (1) were RCTs; (2) acute or chronic stroke patients with swallowing dysfunction; (3) used some kind of noninvasive stimulation; (4) had complete data on outcome indicators; (5) the intervention was any of NmeS, PES, rTMS, and tDCS in the experimental group, and only routine swallowing rehabilitation intervention or no intervention in the control group; (6) testing at least one of the metrics: FDS, FoiS, VFSS, DSRS, DSR, DS, PAS, DOSS, BI, SWAL-QOL, ASHA NOMS, CDS, and MASA.

Studies were excluded if they: (1) were non-RCTs (2) were experimental animal studies, review-type literature, conference reports, case reports, letters, and repetitively published literature, etc.; (3) full text was not available; (4) experimental outcome data were incomplete or data metrics could not be extracted; (5) relevant metrics of interest to this study were not reported; (6) included patients whose dysphagia was caused by other diseases or who had a history of previous dysphagia; (7) non-core journal literature in Chinese published literature.

### 2.4 Four non-invasive stimulations

The type, frequency, and duration of the stimuli used in each study are shown in Table 1.

**Table 1:** Stimulus features of the included studies.

Study	Stimulus type	Stimulus modelity	Stimulus site	Stimulus frequency and duration
Lim et al. (2014)	NmeS	Pulse width 300 μs, pulse frequency 80 Hz, inter stimulus intervals 100 μs, intensity 7 mA and 9 mA	1-between the digastrics muscle and the hyoid bone and between the hyoid bone and the thyroid cartilage 2-between the thyroid cartilage and the cricoids cartilage and vertically under the cricoid cartilage	30 min, once/day, 5 days/week, 2 weeks
Toyama et al. (2014)	NmeS	Fixed pulse duration 50 us, frequency 50 Hz	The bilateral geniohyoid, mylohyoid/anterior belly of the digastric, and thyrohyoid muscles	40 min, once/day, 5 days/week, 8 weeks
Zhang et al. (2016)	NmeS	Pulse width 100 ms, pulse time 10 s, frequency 120 Hz, intensity 0 to 60 mA	Skin of the anterior belly of the digastric muscle in the submental region above the hyoid bone	20 min, twice/day, 5 days/week, 4 weeks
Chen et al. (2022)	NmeS	Pulse width 300 μs, pulse frequency 80 Hz, intensity 0–25 mA	Above the hyoid bone and below the thyroid notch	30 min, once/day, 5 days/week, 4 weeks
Bengisu et al. (2024)	NmeS	Fixed pulse duration 700 μs, frequency 80 Hz	Mylohyoid muscle and thyroid muscle	40 min, once/day, 5 days/week, 2 weeks
Jayasekeran et al. (2010)	PES	0.2 ms pulses, 280 V, 5 Hz frequency, intensity 75% of maximal tolerated	Trans nasally or trans orally	10 min, once/day, 3 days
Vasant et al. (2016)	PES	0.2 ms pulses, 280 V, 5 Hz frequency, intensity 75% of maximal tolerated	Trans nasally or trans orally, ~14 cm from the incisors or ~15 cm from the nasal flare	10 min, once/day, 3 days
Vasant et al. (2016)	PES	0.2 ms pulses, 280 V, 5 Hz frequency, intensity 75% of maximal tolerated	Trans nasally or trans orally, midpharyngeal level (17 cm from the nasal flare or 15 cm aboral)	10 min, once/day, 3 days
Bath et al. (2016)	PES	1 mA, 5 Hz frequency, intensity 75% of maximal tolerated	Via the nose, aboral depth	10 min, once/day, 3 days
Khedr et al. (2009)	rTMS	3 Hz, 300 pulses, 120% of hand motor threshold intensity	Oesophageal cortical area of the affected hemisphere	10 min, once/day, 5 days
Ünlüer et al. (2019)	rTMS	1 Hz, 1,200 pulses, 90% of the resting motor threshold intensity	The vertex of the cranium	20 min, once/day, 3 days/week, 4 weeks
Jiao et al. (2019)	rTMS	3 Hz, stimulus time 3 s, stimulus interval 17 s, 80% of the resting motor threshold intensity		20 min, once/day, 5 days/week, 2 weeks
Zhong et al. (2021)	rTMS	10 Hz, 250 pulses, 80% of the resting motor threshold intensity	Bilateral pharyngeal motor area of the cerebellum	4 min35s, once/day, 5 days/week, 2 weeks
Suh et al. (2024)	iTBS	Three pulses (60 ms) of stimulation delivered at 50 and 5 Hz TBS train lasting 2 s repeated every 10 s for a total of 200 s, 600 pulses, 80% of the resting motor threshold intensity	Ipsilesional pharyngeal motor cortex	200 s, once/day, 5 sessions
Yang et al. (2012)	tDCS	1 mA	Affected hemisphere that can induce maximal pharyngeal response	20 min, once/day, 5 days/week, 2 weeks
Ahn et al. (2017)	tDCS	1 mA	Bilateral pharyngeal motor cortices	20 min, once/day, 5 days/week, 2 weeks
Farpour et al. (2023)	tDCS	2 mA	Supramarginal gyrus	20 min, once/day, 5 days

#### 2.4.1 Neuromuscular electrical stimulation

NmeS involves applying electrical impulses to muscles to stimulate their contraction. In the context of swallowing disorders, NmeS is typically used to target the muscles responsible for swallowing, such as those in the throat and mouth. These electrical impulses can help enhance muscle strength and coordination, which is particularly useful for individuals with weakened or underactive swallowing muscles. NmeS can improve swallowing function by promoting better synchronization of the muscles involved, leading to improved bolus propulsion and reduced risk of aspiration.

#### 2.4.2 Pharyngeal electrical stimulation

PES is a specific type of electrical stimulation aimed at stimulating the muscles in the pharynx (throat) region to enhance swallowing function. PES involves applying electrical impulses to the area around the pharyngeal muscles, often using external electrodes placed on the skin. This technique is designed to improve the timing and coordination of swallowing by targeting the sensory and motor pathways involved in the process.

#### 2.4.3 Repetitive transcranial magnetic stimulation

rTMS is a non-invasive technique that uses magnetic fields to stimulate specific regions of the brain. In the treatment of swallowing difficulties, rTMS targets the motor cortex areas responsible for controlling the swallowing muscles. By modulating neural activity, rTMS can enhance brain plasticity and improve the function of the muscles involved in swallowing. rTMS can improve swallowing outcomes by increasing cortical excitability and facilitating the neural control of swallowing. This results in better motor coordination, muscle activation, and reduced incidence of aspiration, providing a promising option for individuals with neurogenic dysphagia.

#### 2.4.4 Transcranial direct current stimulation

TDCS involves the application of a low electrical current to the scalp through electrodes, which modulates neuronal activity in specific areas of the brain. In the context of swallowing disorders, tDCS can be targeted to brain regions involved in motor control, including areas responsible for swallowing. The application of tDCS can either enhance or inhibit neural activity, depending on the direction of the current, which can lead to improvements in swallowing function. For individuals with dysphagia due to neurological damage, such as after a stroke, tDCS has been shown to promote neuroplasticity, improve motor function, and restore more efficient swallowing patterns.

### 2.5 Outcomes

Multiple scales are available to assess swallowing function, providing an accurate reflection of the patient’s swallowing ability and the degree of impairment. The Functional Dysphagia Scale (FDS) is used to evaluate the patient’s ability to swallow different food textures, helping to determine the severity of swallowing difficulties. The Functional Oral Intake Scale (FoiS) assesses the level of oral intake, ranging from total dependence on feeding tubes to full independence in eating, reflecting the patient’s swallowing capabilities. The video fluoroscopic swallowing study (VFSS) allows real-time observation of the swallowing process through radiographic imaging, providing a detailed assessment of the safety and function of swallowing. The Dysphagia Severity Rating Scale (DSRS) evaluates the severity of swallowing disorders based on clinical symptoms, ranging from mild to severe. The Penetration Aspiration Scale (PAS) assesses the extent of food or liquid entering the airway, reflecting the safety of swallowing. Finally, the Dysphagia Outcome and Severity Scale (DOSS) helps to grade the patient’s swallowing impairment by comprehensively evaluating swallowing function and its impact on daily life. These scales together provide a comprehensive tool for assessing swallowing function.

When assessing the impact of swallowing disorders on the patient’s quality of life, several scales provide valuable reference tools for clinical practice. The Barthel index (BI) is primarily used to assess the patient’s ability to perform activities of daily living, such as eating, dressing, and bathing, reflecting the impact of swallowing function on daily life. The Swallowing Quality of Life (SWAL-QOL) scale is specifically designed to assess the quality of life changes due to swallowing disorders, covering aspects such as emotional, social, and physical health. The American Speech-Language-Hearing Association National Outcome Measurement System (ASHA NOMS) provides a standardized framework for evaluating the impact of swallowing disorders on the patient’s quality of life and rehabilitation progress. The Clinical Dysphagia Scale (CDS) offers valuable information for clinicians by comprehensively assessing swallowing disorders and their impact on quality of life. Finally, the Modified Mann Assessment of Swallowing Ability (MASA) evaluates both swallowing function and the relationship between swallowing ability and quality of life. These scales together help us better understand the comprehensive impact of swallowing disorders on the patient’s quality of life.

### 2.6 Data collection

Two researchers (HL and XL) screened the collected literature by importing it into EndNote 20 software according to the search strategy developed. All duplicates were first excluded, followed by reading the titles and abstracts for initial screening. Subsequently, the remaining literature was screened in more depth by reading the full text in detail according to the preset inclusion and exclusion criteria. Next, HL and XL cross-checked their respective screening results and literature in agreement would be included in the study. In case of disagreement, a third researcher (HS) would be consulted and after discussion and agreement, the literature to be included in the study would be finalized. This process ensures the rigor and reliability of literature screening.

For eligible experiments, data from the included literature were independently extracted and generalized for risk of risk bias by two trained researchers (HL and XL) using a standardized data extraction form. The extracted data mainly included (1) basic information about the included literature (first author, year of publication, country, etc.); (2) demographic characteristics of the subjects (number of experimental and control groups, age, sex, and duration of the disease); (3) details of the interventions (type of intervention, intensity, duration, and frequency); and (4) outcome metrics (mean and standard deviation; selected outcome metrics included scales describing swallowing function and QoL. The scales of swallowing function such as FDS, FoiS, VFSS, DSRS, PAS and DOS. QoL scales such as BI, SWAL-QOL, ASHA NOMS, CDS, and MASA). For studies where results were presented graphically without numerical summaries, numerical data were extracted for analysis using a validated plot digitizing tool (GetData 2.22). We contacted the authors of the articles for information when necessary.

### 2.7 Risk of bias of the systematic review

Based on the Cochrane 5.1 version of the risk of bias assessment tool, which covers seven domains (random sequence generation, allocation concealment, blinding of participants and personnel, blinding of outcome assessor, incomplete data outcome, selective reporting, and other bias), and two researchers (HL and XL) assessed risk of bias (ROB) for all eligible studies. The results of the assessment in each area were categorized as unclear, low risk, and high risk. Based on these assessments, we categorized the overall risk of bias for each study as (1) low ROB: there were no areas assessed as high risk, and there may have been areas assessed as unclear but fewer than three; (2) medium ROB: there was an area assessed as high risk but no more than one; or there were no areas of high risk but there were more than three assessed as unclear; and (3) high ROB: all areas other than the above were categorized as high risk. This systematic approach to assessment ensured a comprehensive analysis of study quality.

### 2.8 Statistical analysis

In this study, data were analyzed by META using STATA 17.0 software (StataCorp LLC, College Station, TX, United States), with outcome indicators as continuous variables. This NMA integrated the pre- and post-changes in the experimental and control groups to systematically assess the effects of different non-invasive stimuli (including NmeS, PES, rTMS and tDCS) on swallowing function and QoL indicators, and to accurately assess the effects of these interventions, we calculated the standardized mean difference (SMD) of each indicator and its 95% confidence interval (CI), with a uniform adjustment of the baseline to *α* = 0.05, and combined effect estimates based on a random-effects model to account for heterogeneity between studies in terms of participant characteristics and intervention modalities. Heterogeneity was quantified using the *I*^2^ statistic and Cochran’s *Q* test. Relationships between different non-invasive stimuli were visualized by means of network diagrams, where lines connecting nodes represent direct comparisons between different non-invasive stimuli. The size of the nodes and the thickness of the connecting lines were proportional to the number of studies that included that comparison, and this graphical presentation visualized the relative strengths of the interventions and their positions in the network. In addition, the plotted network contributions further quantify the contribution of each direct comparison to the overall network, helping to analyze the influence of each intervention across the network. Additionally, to assess publication bias in the study, corrected comparison funnel plots were used to analyze publication bias for the primary outcome metrics. Finally, the probability of being the best intervention was calculated using a cumulative lower surface of the ranking curve (SUCRA) approach.

## 3 Result

### 3.1 Study selection

**Figure 1:**
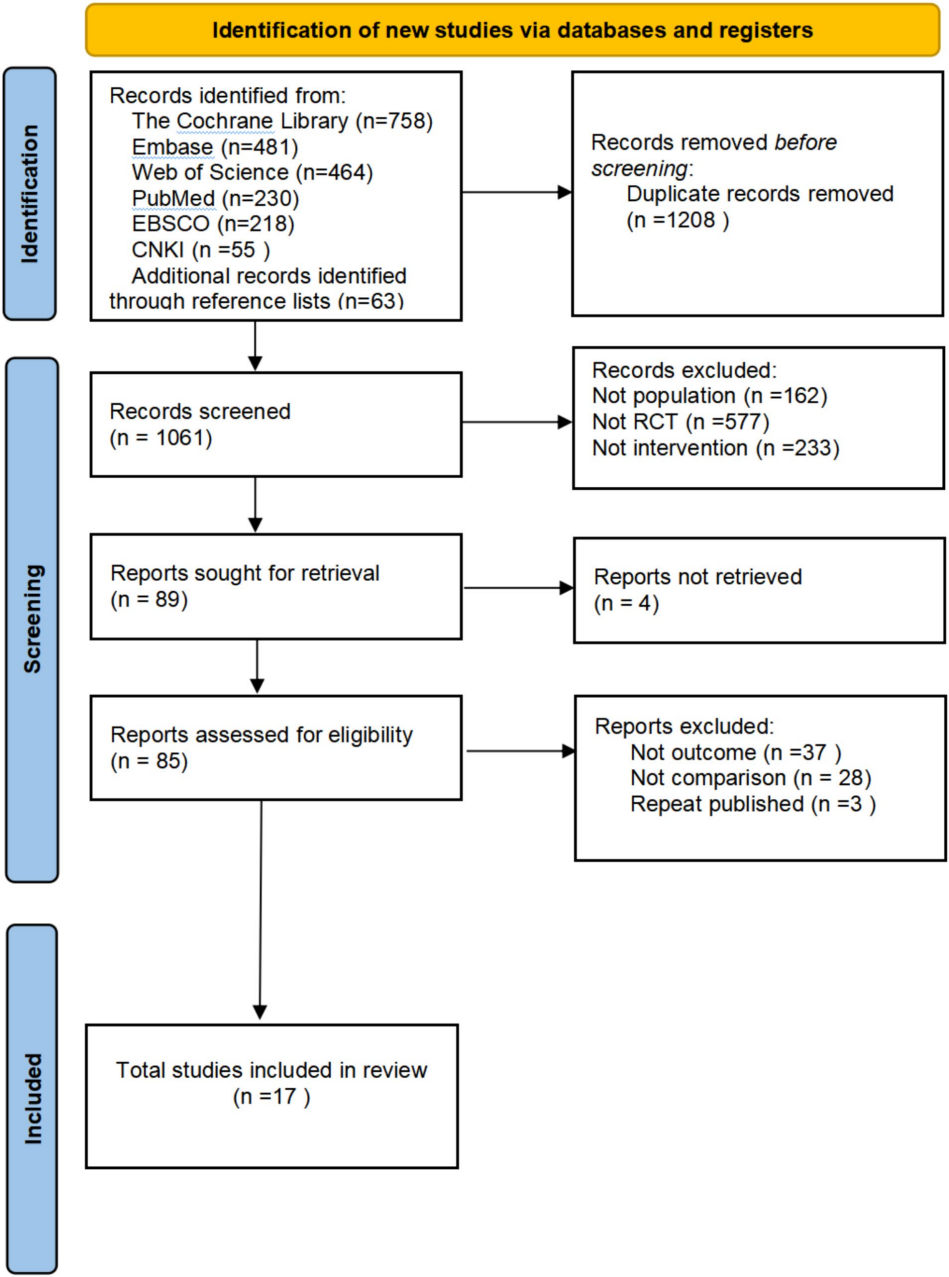
Literature search flowchart.

The flowchart for study selection is shown in Figure 1. A total of 2,269 potentially eligible articles were identified. After removing 1,208 duplicate articles, 1,061 articles remained to be screened. Seventeen randomized experiments were finalized by screening titles and abstracts, removing 972 articles, and obtaining and reading 85 full-text articles. Four different non-invasive stimulation methods were evaluated.

### 3.2 Features of the included studies

Seventeen studies were finally included, and the basic characteristics of all the included studies are detailed in Table 1. These studies were published between 2008 and 2024 and were conducted in China, the United Kingdom, Korea, Japan, Turkey, and Iran. A total of 788 stroke patients were included in this study, 405 in the experimental group and 383 in the control group. Demographic data reported included age, gender, disease duration, and stroke type. Rehabilitation methods included NmeS, PES, rTMS, and tDCS. The mean duration of treatment for the different noninvasive stimulation interventions was 2.3 weeks, with 58.8% of the studies reporting interventions lasting longer than 2 weeks.

Regarding the reported outcome indicators, the outcome indicators for swallowing function were FDS, FoiS, VFSS, DSRS, PAS, and DOSS, and the outcome indicators for QoL were BI, SWAL-QOL, ASHA NOMS, CDS, and MASA. The basic characteristics of all included studies are detailed in Table 2.

**Table 2:** Basic features of the included studies.

Study	Country	Stroke stage	Group	Sample size (M/F)	Age (mean ± SD)	Days from onset (mean ± SD)	Intervention category	Intervention frequency and duration	Outcome measures
Lim et al. (2014)	Korea	Acute	TG	18 (12/6)	66.3 ± 15.4	37.3 ± 16.1	NmeS + RTD	NmeS: 30 min, once/day, 5 days/week, 2 weeks	FDS, ASHA NOMS
			CG	15 (9/6)	62.5 ± 8.2	34.4 ± 10.1	RTD	RTD: 30 min, once/day, 5 days/week, 4 weeks	
Toyama et al. (2014)	Japan	Acute and chronic	TG	12 (12/0)	63.6 ± 21.4	176.4 ± 181.3	NmeS + RTD	rTMS: 20 min, 10 sessions	FoiS, VDS
			CG	14 (10/4)	67.2 ± 13.7	102.9 ± 74.2	RTD	RTD: 40 min, once/day, 5 days/week, 8 weeks	
Zhang et al. (2016)	China	Acute	TG	28 (16/12)	61.3 ± 7.1	22.1 ± 4.0	NmeS + RTD	NmeS: 20 min, twice/day, 5 days/week, 4 weeks	FoiS, SWAL-QOL
			CG	27 (17/10)	62.6 ± 8.7	21.3 ± 4.1	RTD		
Chen et al. (2022)	China	Acute	TG	50 (26/24)	67.17 ± 4.28	20.76 ± 5.78	NmeS + RSR	NmeS: 30 min, once/day, 5 days/week, 4 weeks	VFSS
			CG	50 (28/22)	67.59 ± 4.50	21.44 ± 5.45	RSR	RSR: once/day, 5 days/week, 4 weeks	
Bengisu et al. (2024)	Turkey	Acute	TG	10 (7/3)	66.9 ± 12.5		NmeS + RTD	NmeS: 40 min, once/day, 5 days/week, 2 weeks	DSRS
			CG	10 (7/3)	68.0 ± 10.5		Sham NmeS + RTD	RDT: 60 min, once/day, 5 days/week, 2 weeks	
Jayasekeran et al. (2010)	UK	Acute	TG	16	75 ± 2.7		PES (5 Hz)	PES: 10 min, once/day, 3 days	DSRS
			CG	12	74 ± 2.3		Sham PES		
Vasant et al. (2016)	UK	Acute	TG	8 (5/3)	58.6 ± 13.42		PES (5 Hz)	PES: 10 min, once/day, 3 days	DSRS
			CG	8 (5/3)	70.5 ± 11.8		Sham PES		
Vasant et al. (2016)	UK	Acute	TG	18 (9/9)	71 (56, 79)	16 ± 10.37	PES (5 Hz)	PES: 10 min, once/day, 3 days	DSRS
			CG	18 (13/5)	71 (61, 78)	11 ± 7.41	Sham PES		
Bath et al. (2016)	UK	Acute	TG	87 (48/39)	74.0 ± 9.9	77/9/0	PES (5 Hz) + SC	PES: 10 min, once/day, 3 days	PAS, BI
			CG	75 (46/29)	74.9 ± 12.6	66/8/1	Sham PES + SC		
Khedr et al. (2009)	UK	Acute	TG	14	58.9 ± 11.7		rTMS (3 Hz)	rTMS: 10 min, once/day, 5 days	DSRS, BI
			CG	12	56.2 ± 13.4		Sham rTMS		
Ünlüer et al. (2019)	Turkey	Acute	TG	15 (9/6)	67.80 ± 11.88	14/1/0	rTMS (1 Hz) + RTD	rTMS: 20 min, once/day, 3 days/week, 4 weeks	PAS, SWAL-QOL
			CG	13 (7/6)	69.31 ± 12.89	12/1/0	RTD	RTD: 30–45 min, once/day, 3 days/week, 4 weeks	
Jiao et al. (2019)	China	Acute	TG	30 (14/16)	64.5 ± 4.3		rTMS (3 Hz) + BR	rTMS: 20 min, once/day, 5 days/week, 2 weeks	PAS, CDS
			CG	30 (19/11)	65.2 ± 5.1		BR	BR: twice/day, 5 days/week, 2 weeks	
Zhong et al. (2021)	China	Acute	TG	41 (24/17)	63.61 ± 9.674	27/14/0	rTMS (10 Hz) + RSR	rTMS: 4 min35 s, once/day, 5 days/week, 2 weeks	PAS
			CG	43 (21/22)	62.81 ± 11.49	26/17/0	Sham rTMS + RSR	RSR: 30 min, once/day, 5 days/week, 2 weeks	
Suh et al. (2024)	Korea	Acute and chronic	TG	14 (7/7)	64.39 ± 16.60	9/5/0	iTBS + RTD	iTBS: 200 s, once/day, 5 sessions	PAS
			CG	14 (9/5)	68.64 ± 12.83	10/4/0	Sham iTBS + RTD	RDT: 30 min, once/day, 5 sessions	
Yang et al. (2012)	Korea	Acute	TG	9 (6/3)	70.44 ± 12.59		tDCS + ST	tDCS: 20 min, once/day, 5 days/week, 2 weeks	FDS
			CG	7 (3/4)	70.57 ± 8.46		Sham tDCS + ST	ST: 30 min, once/day, 5 days/week, 2 weeks	
Ahn et al. (2017)	Korea	Chronic	TG	13 (9/4)	61.62 ± 10.28	5/8/0	tDCS + RTD	tDCS: 20 min, once/day, 5 days/week, 2 weeks	DOSS
			CG	13 (6/7)	66.38 ± 10.67	11/2/0	Sham tDCS + RTD		
Farpour et al. (2023)	Iran	Acute	TG	22 (13/9)	65.32 ± 16.34		tDCS + BT	tDCS: 20 min, once/day, 5 days	FoiS, MASA
			CG	22 (10/12)	70.68 ± 16.33		Sham tDCS + BT		

TG, test group; CG, control group.

### 3.3 Risk of bias assessment

**Figure 2:**
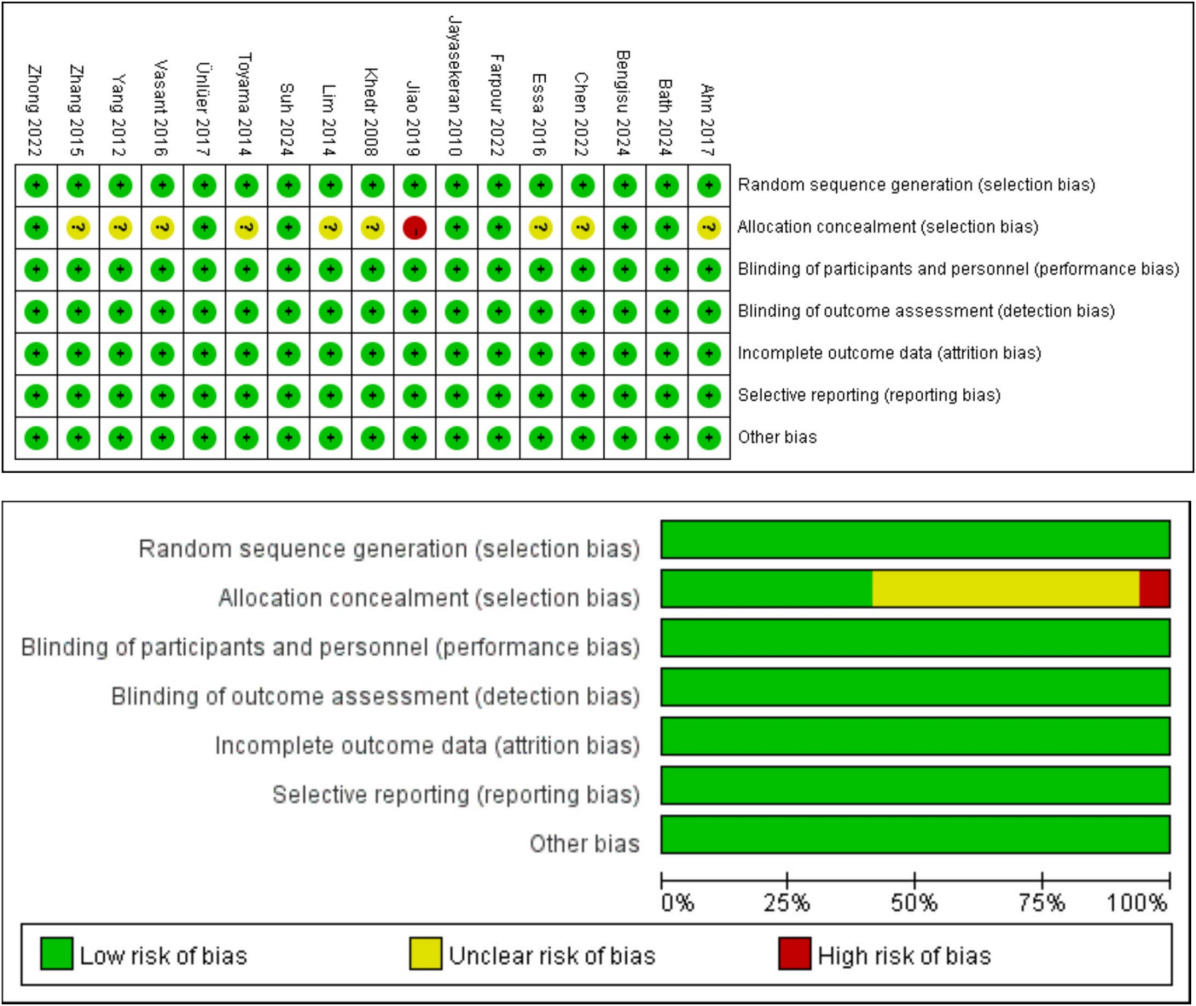
Graph of the results of the quality risk bias assessment of the literature included in the study.

Of the 17 articles, a total of 17 articles mentioned random allocation, of which 10 specified the method of random allocation; eight stated allocation concealment, starting with one at high risk; 17 reported blinding; 17 reported blinding of outcome assessment; 17 studies demonstrated low risk of selective reporting; and all articles were free of other biases. In summary, 16 articles were judged to have a low ROB and one a medium ROB. When conducting the risk shift analysis, we noticed that the age change of the study population selected in the article was relatively small, and there was no significant fluctuation in the age range of the sample population. Therefore, the potential influence of age on the study findings is more consistent. Meanwhile, the main results reported focus on swallowing function and QoL, and these results provide a clear focus for the analysis in this paper. The reliability of the findings and the low risk of bias helped to reduce interference with risk assessment. The results of the literature quality assessment are detailed in Table 3 and Figure 2.

**Table 3:** Evaluation results of literature quality risk bias of included studies.

Inclusion of literature	Random sequence generation	Allocation concealment	Blinding of participants and personnel	Blinding of outcome assessment	Incomplete outcome data	Selective reporting	Other bias
Lim et al. (2014)	L	U	L	L	L	L	L
Toyama et al. (2014)	L	U	L	L	L	L	L
Zhang et al. (2016)	L	U	L	L	L	L	L
Chen et al. (2022)	L	U	L	L	L	L	L
Bengisu et al. (2024)	L	L	L	L	L	L	L
Jayasekeran et al. (2010)	L	L	L	L	L	L	L
Vasant et al. (2016)	L	U	L	L	L	L	L
Vasant et al. (2016)	L	U	L	L	L	L	L
Bath et al. (2016)	L	L	L	L	L	L	L
Khedr et al. (2009)	L	U	L	L	L	L	L
Ünlüer et al. (2019)	L	L	L	L	L	L	L
Jiao et al. (2019)	L	H	L	L	L	L	L
Zhong et al. (2021)	L	L	L	L	L	L	L
Suh et al. (2024)	L	L	L	L	L	L	L
Yang et al. (2012)	L	U	L	L	L	L	L
Ahn et al. (2017)	L	U	L	L	L	L	L
Farpour et al. (2023)	L	L	L	L	L	L	L

L, low risk of bias; U, unclear risk of bias; H, high risk of bias.

### 3.4 Network meta-analysis

#### 3.4.1 Network diagram of included studies

**Figure 3:**
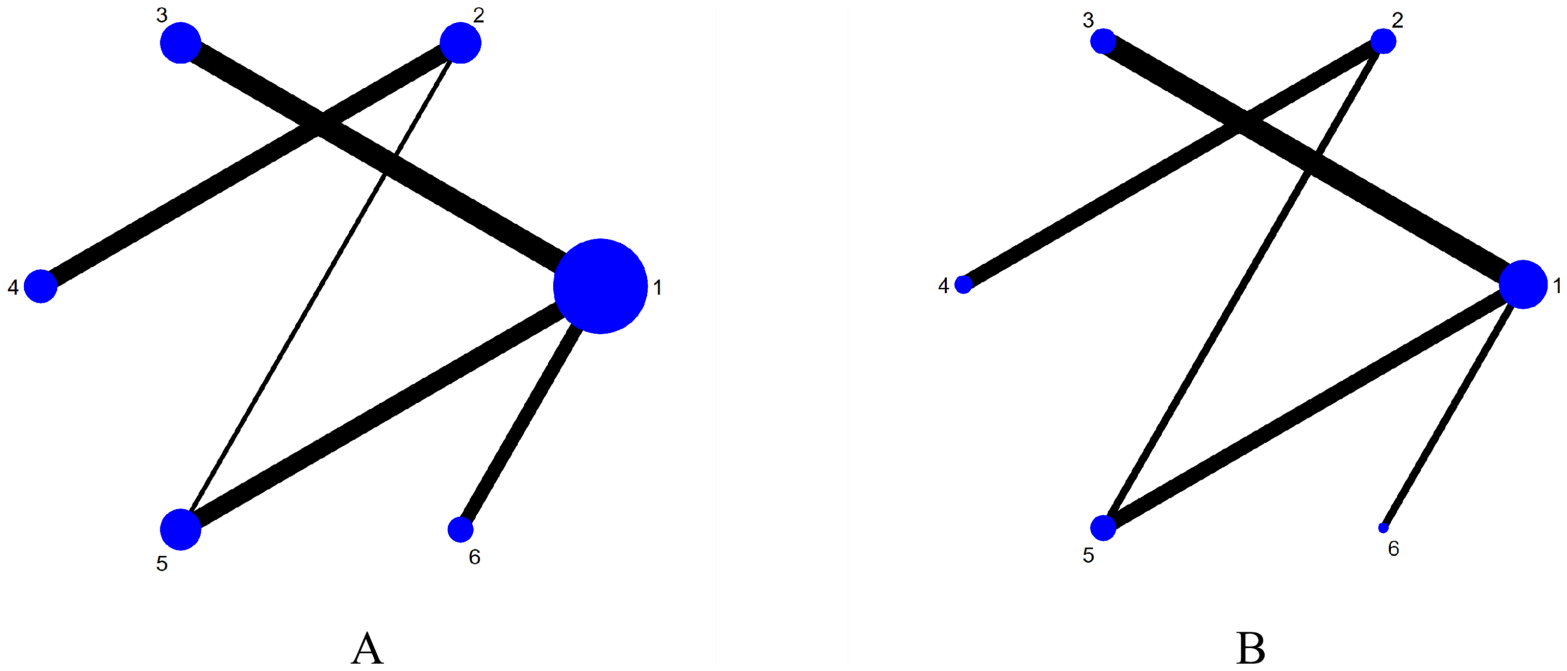
Network plot of outcome indicators (**A** is swallowing function, **B** is is QoL). 1 = Routine swallowing rehabilitation; 2 = No intervention; 3 = NmeS; 4 = PES; 5 = rTMS; 6 = tDCS.

The six dots in the figure represent the six interventions. The straight lines between the dots represent the existence of direct comparisons between interventions. The size of the dots reflects the number of studies included in each group, with larger dots representing a greater number of studies. The thickness of the line represents the number of direct comparisons between the two interventions. The outcome indicators are all four interventions (including the control group) and include the same interventions. The experimental group interventions included NmeS, PES, rTMS, and tDCS; the control group was routine swallowing rehabilitation only or no intervention, with NmeS being the most widely studied intervention and tDCS being less studied. The Network diagram of the outcome metrics is detailed in Figure 3.

#### 3.4.2 Direct pairwise meta-analyses

##### 3.4.2.1 Swallowing function metrics

**Figure 4:**
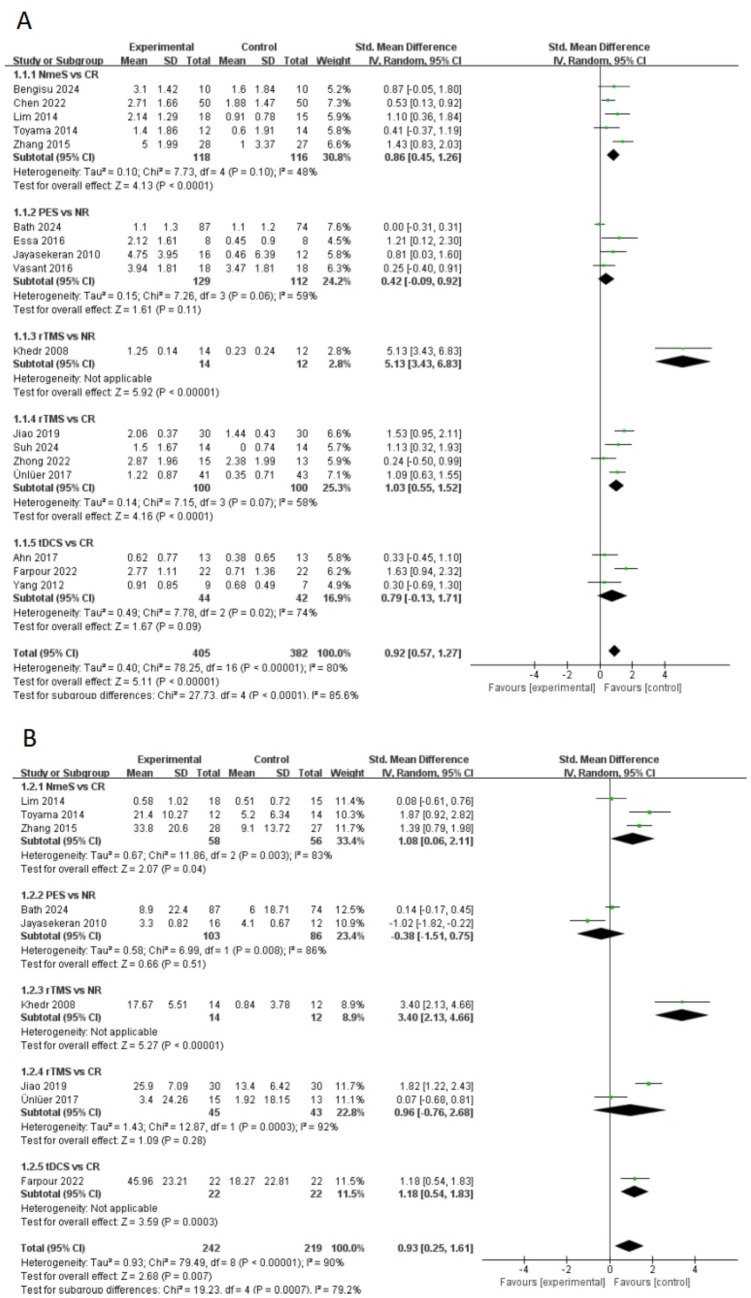
Forest plots (**A** is swallowing function, **B** is QoL).

A total of 17 papers included swallowing function indicators. A pairwise meta-analysis was first conducted, and the forest plot of swallowing function was shown in Figure 4A. NmeS [SMD = 0.86, 95% CI (0.45, 1.26), *p* < 0.0001, *I*^2^ = 48%] and rTMS [SMD = 1.03, 95% CI (0.55, 1.52), *p* < 0.0001, *I*^2^ = 58%] showed significant effects in improving swallowing function compared with CR, with moderate heterogeneity. rTMS showed a similarly significant effect that improved swallowing function compared with NR [SMD = 5.13, 95% CI (3.43, 6.83), *p* < 0.00001]. tDCS, although effective in improving swallowing function, showed no significant effect compared with CR [SMD = 0.79, 95% CI (−0.13, 1.71), *p* > 0.05, *I*^2^ = 74%], suggesting that the results varied across studies. Similarly, the effect of PES on swallowing function compared with NR was nonsignificant [SMD = 0.42, 95% CI (−0.09, 0.92), *p* > 0.05, *I*^2^ = 59%], with moderate heterogeneity.

##### 3.4.2.2 QoL indicators

A total of nine papers included QoL indicators. The forest plot of QoL was shown in Figure 4B. NmeS [SMD = 1.08, 95% CI (0.06, 2.11), *p* < 0.05, *I*^2^ = 83%] and tDCS [SMD = 1.18, 95% CI (0.54, 1.83), *p* < 0.001] were significantly effective for improving QoL compared to CR. Compared to NR, rTMS [SMD = 3.40, 95% CI (2.13, 4.66), *p* < 0.00001] showed significant effects in improving QoL. rTMS [SMD = 0.966, 95% CI (−0.76, 2.68), *p* > 0.05, *I*^2^ = 92%] had no significant effect on QoL’s improvement compared to CR. Although PES [SMD = −0.38, 95% CI (−1.51, 0.75), *p* > 0.05, *I*^2^ = 86%] was effective in improving QoL, it was not significant compared with NR and showed high heterogeneity.

#### 3.4.3 Effects of four non-invasive stimulations on swallowing function and QoL

**Figure 5:**
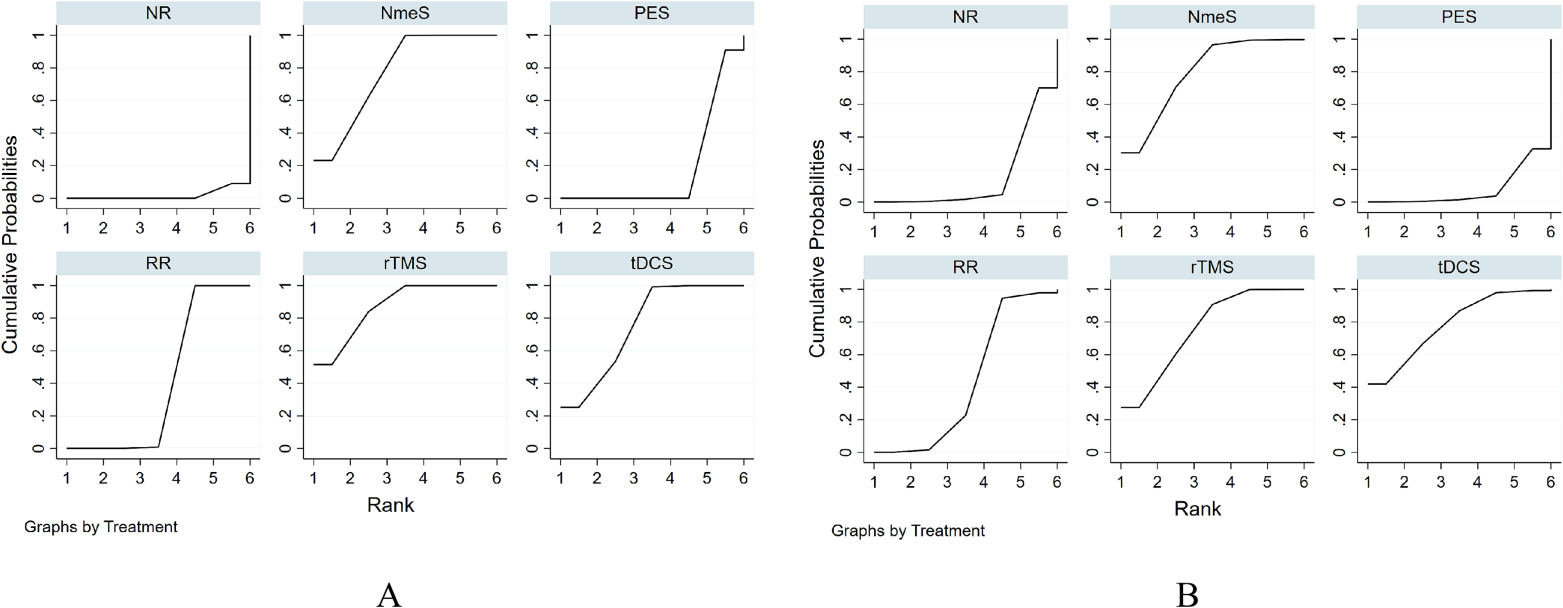
Ranking of intervention effects for outcome indicators (**A** is swallowing function, **B** is QoL).

For swallowing function indicators, rTMS [SMD = 5.10, 95% CI (3.20, 7.01), *p* < 0.0001], tDCS [SMD = 4.90, 95% CI (2.81, 6.98), *p* < 0.0001], NmeS [SMD = 4.94, 95% CI (2.91, 6.97), *p* < 0.0001], RR [SMD = 4.08, 95% CI (2.10, 6.05), *p* < 0.05] demonstrated improvements in swallowing function metrics compared with NR. The improvement in PES [SMD = 0.36, 95% CI (−0.18, 0.91), *p* > 0.05] was not significant. The effectiveness of the four noninvasive stimuli on swallowing function in stroke patients was ranked as rTMS [(SUCRA) = 87.3], tDCS [(SUCRA) = 75.3], and NmeS [(SUCRA) = 77.3] were superior to RR with conventional swallowing rehabilitation intervention [(SUCRA) = 40.1] or NR without other interventions [(SUCRA) = 1.8], PES [(SUCRA) = 18.2] were superior to NR without other interventions [(SUCRA) = 1.8]. See Figure 5A and Tables 4, 5 for details.

**Table 4:** Network meta-analysis matrix of outcomes.

Swallowing function					
tDCS	0.21 (−0.64, 1.05)	−0.82 (−1.49, −0.15)	−4.54 (−6.69, −2.38)	0.04 (−0.78, 0.86)	−4.90 (−6.98, −2.81)
−0.21 (−1.05, 0.64)	rTMS	−1.03 (−1.55, −0.51)	−4.74 (−6.72, −2.76)	−0.17 (−0.87, 0.54)	−5.10 (−7.01, −3.20)
0.82 (0.15, 1.49)	1.03 (0.51, 1.55)	RR	−3.71 (−5.76, −1.67)	0.86 (0.39, 1.34)	−4.08 (−6.05, −2.10)
4.54 (2.38, 6.69)	4.74 (2.76, 6.72)	3.71 (1.67, 5.76)	PES	4.58 (2.48, 6.68)	−0.36 (−0.91, 0.18)
−0.04 (−0.86, 0.78)	0.17 (−0.54, 0.87)	−0.86 (−1.34, −0.39)	−4.58 (−6.68, −2.48)	NmeS	−4.94 (−6.97, −2.91)
**4.90 (2.81, 6.98)**	**5.10 (3.20, 7.01)**	**4.08 (2.10, 6.05)**	0.36 (−0.18, 0.91)	**4.94 (2.91, 6.97)**	NR
QoL					
tDCS	−0.21 (−2.70, 2.27)	−1.18 (−3.21, 0.85)	−3.95 (−7.63, −0.28)	−0.09 (−2.44, 2.26)	−3.60 (−6.99, −0.21)
0.21 (−2.27, 2.70)	rTMS	−0.97 (−2.41, 0.47)	−3.74 (−6.45, −1.03)	0.12 (−1.75, 1.99)	−3.38 (−5.69, −1.08)
1.18 (−0.85, 3.21)	0.97 (−0.47, 2.41)	RR	−2.77 (−5.84, 0.29)	1.09 (−0.10, 2.28)	−2.42 (−5.13, 0.30)
3.95 (0.28, 7.63)	3.74 (1.03, 6.45)	2.77 (−0.29, 5.84)	PES	3.86 (0.57, 7.15)	0.35 (−1.07, 1.78)
0.09 (−2.26, 2.44)	−0.12 (−1.99, 1.75)	−1.09 (−2.28, 0.10)	−3.86 (−7.15, −0.57)	NmeS	−3.51 (−6.47, −0.54)
**3.60 (0.21, 6.99)**	**3.38 (1.08, 5.69)**	2.42 (−0.30, 5.13)	−0.35 (−1.78, 1.07)	**3.51 (0.54, 6.47)**	NR

Bold indicates a significant difference between the two comparisons.

**Table 5:** Ranking of the probability of improving swallowing function and QoL class in stroke patients by four non-invasive stimulations.

Treatment	Swallowing function		QoL	
	SUCRA (%)	Rank	SUCRA (%)	Rank
RR	40.1	4	43.4	4
NR	1.8	6	15.4	5
NmeS	77.3	3	79.3	1
PES	18.2	5	7.7	6
rTMS	87.3	1	75.7	3
tDCS	75.3	2	78.6	2

For QoL indicators, NmeS [SMD = 3.51, 95% CI (0.54, 6.47), *p* < 0.0001], tDCS [SMD = 3.60, 95% CI (0.21, 6.99), *p* < 0.0001], rTMS [SMD = 3.38, 95% CI (1.08, 5.69), *p* < 0.0001] demonstrated improvements in QoL compared with NR. The improvements in PES [SMD = −0.35, 95% CI (−1.78, 1.07), *p* > 0.05] and RR [SMD =2.42, 95% CI (−0.30, 5.13), *p* > 0.05] were not significant. The effectiveness of the four noninvasive stimuli on the QoL of stroke patients was ranked as NmeS [(SUCRA) = 79.3], tDCS [(SUCRA) = 78.6], and rTMS [(SUCRA) = 75.7] were superior to the RR [(SUCRA) = 43.4], which had a conventional swallowing rehabilitation intervention, or to the NR without other interventions [(SUCRA) = 15.4], PES [(SUCRA) = 7.7] were not superior to the control group. See Figure 5B and Tables 4, 5 for details.

### 3.5 Publication bias test

**Figure 6:**
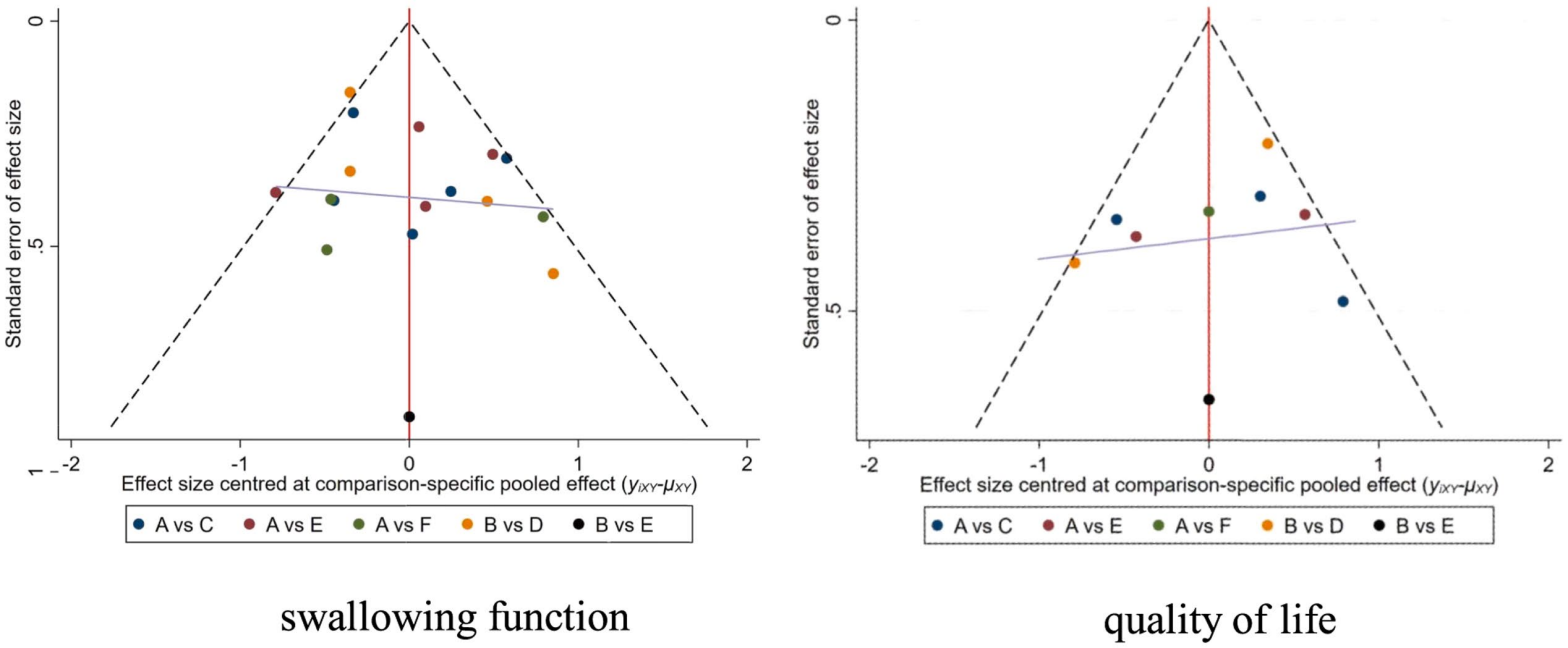
Corrected comparison funnel plot for outcome indicators. A = Routine swallowing rehabilitation; B = No intervention; C = NmeS; D = PES; E = rTMS; F = tDCS.

For studies included in the reticulated META analysis, small-sample effect estimates and publication bias tests were performed using corrected-comparison funnel plots. The included studies were largely symmetrical, suggesting that there was no small-sample effect in the current study, and no significant publication bias was found. See Figure 6 for details.

### 3.6 Heterogeneity handling

**Figure 7:**
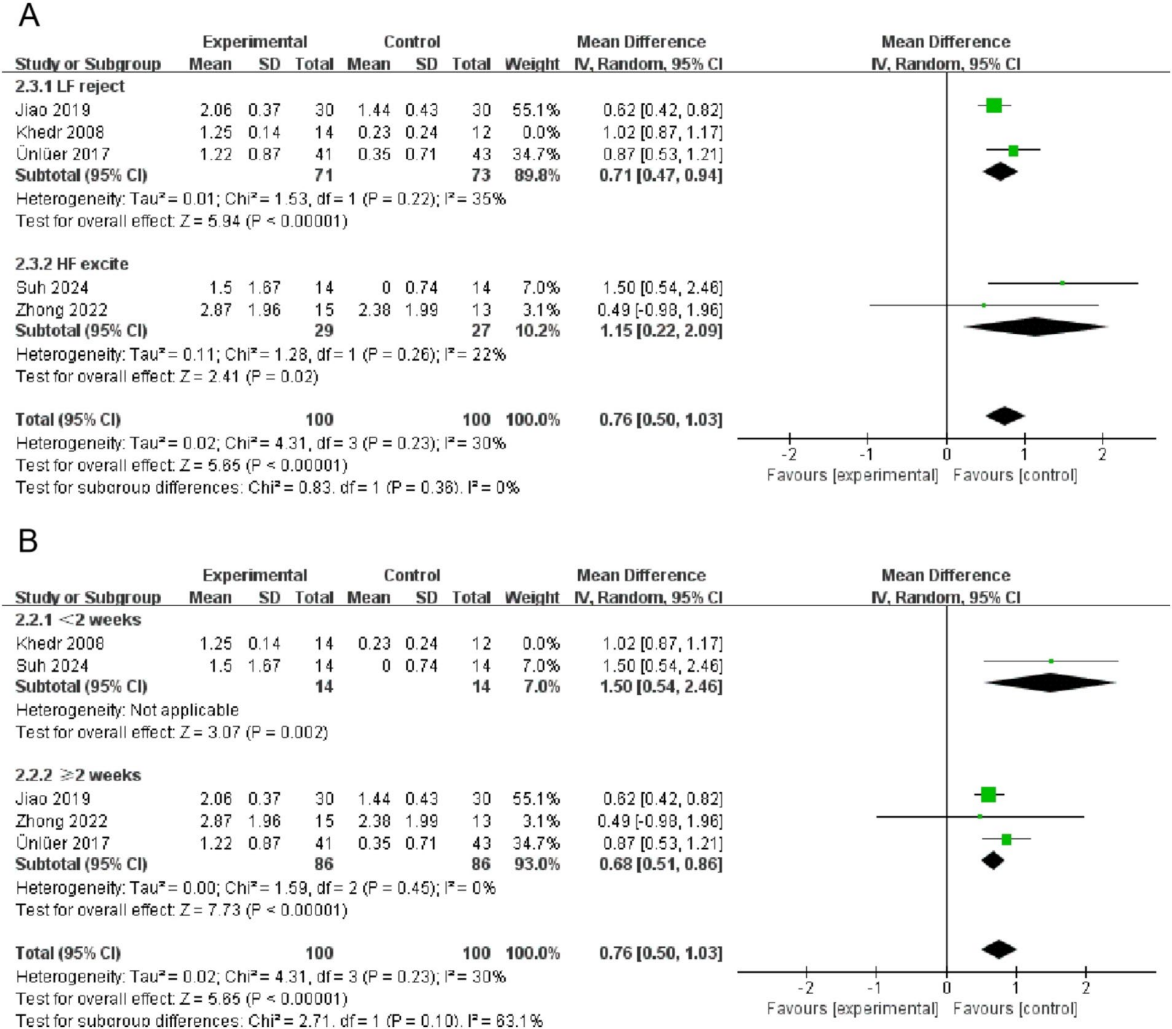
Subgroup forest plot for the swallowing function indicator in the rTMS group (**A** is intervention frequency, **B** is intervention duration).

Significant heterogeneity was observed across numerous intervention measures. Despite the implementation of stringent inclusion criteria, a certain degree of heterogeneity was an inevitable outcome due to differences in the samples of subjects included and methodological discrepancies. Further investigation revealed that the primary contributors to high heterogeneity were the varying frequencies and durations of the interventions. To address this issue, we conducted subgroup analyses of the swallowing function indicators for the rTMS group, stratified by intervention frequency and duration, as shown in Figure 7. Given that the number of studies included in the subgroup analysis did not exceed five, a fixed-effects model was utilized for the analysis. The results indicated a significant reduction in heterogeneity. Observations also suggested that the high heterogeneity in swallowing function indicators for the tDCS group was likewise attributed to differences in intervention frequency and duration. Similarly, heterogeneity in QoL indicators for the NmeS and rTMS groups was due to varying intervention frequencies and durations. However, for the PES group indicators, no clear sources of heterogeneity were identified. We acknowledge this as one of the limitations of our study, which will be further discussed in the limitations section of the discussion.

## 4 Discussion

### 4.1 Analysis of the efficacy of four non-invasive stimuli on swallowing function in stroke patients

In this study, we evaluated the effects of four non-invasive stimuli (NmeS, PES, rTMS, and tDCS) on swallowing function in stroke patients and found that all of these stimulation methods significantly outperformed the control group in improving swallowing function, with all results showing statistical significance. This suggests that non-invasive stimulation can significantly improve swallowing function in stroke patients and may offer new options for relevant clinical interventions. When comparing the effectiveness of different stimulation methods, rTMS [SMD = 5.10, 95% CI (3.20, 7.01), *p* < 0.0001, SUCRA = 87.3] showed the best results. Notably, PES [SMD = 0.36, 95% CI (−0.18, 0.91), *p* > 0.05, SUCRA = 18.2] showed limited improvement in swallowing function, especially when compared with the NR group. It is consistent with some of the results in the literature (Liu et al., 2024), suggesting that it may need to be carefully selected for clinical application. According to the study by Zhang et al. (2022) and Zhang et al. (2023a, 2023b, 2023c), mPES has demonstrated good results in both healthy individuals and patients with neurogenic dysphagia. However, the findings of this study may differ due to variations in inclusion and exclusion criteria or differences in PES parameters.

rTMS showed significant clinical benefits in improving neurological function and swallowing ability, as pointed out by Bath et al. (2018), which is in line with the results of our study consistent with the results of our study. And the study by Yang et al. (2012) also confirmed the effectiveness of tDCS in promoting swallowing function, especially in acute stroke patients. In contrast, the effect of PES was less consistent across studies, which may be related to different implementation protocols and individual patient differences (Suntrup-Krueger et al., 2023).

Regarding the assessment of swallowing function using scales, FDS assesses dysphagia severity through patient self-report, while the FoiS focuses on oral intake and dietary changes. The VFSS is an objective method that uses fluoroscopy to evaluate swallowing structure and function, commonly used to assess safety and efficacy. Both the DSRS and DOSS evaluate dysphagia severity and treatment outcomes. The PAS quantifies the degree of food aspiration during swallowing, serving as a tool for swallowing safety assessment. These scales evaluate swallowing function through subjective reports, objective observations, and quantified scores, aiding in treatment efficacy assessment. Researchers select different scales based on study design. The FDS and FoiS are straightforward but may be influenced by patient subjectivity. The VFSS is the gold standard for diagnosing dysphagia but requires specific equipment and expertise. The DSRS and DOSS demand skilled evaluators, and variability among evaluators may introduce bias. The PAS, while a quantified tool for swallowing safety, requires instrumental examination. Each scale has its strengths and weaknesses, and the appropriate tool should be chosen based on clinical context.

### 4.2 Analysis of the efficacy of four non-invasive stimuli on the QoL of stroke patients

The results showed that rTMS, tDCS and NmeS showed significant improvement in patients’ QoL (*p* < 0.05). In terms of effectiveness ranking, NmeS [SMD = 3.51, 95% CI (0.54, 6.47), *p* < 0.0001, SUCRA = 79.3], tDCS [SMD = 3.60, 95% CI (0.21, 6.99), *p* < 0.0001, SUCRA = 78.6], and rTMS [SMD = 3.38, 95% CI (1.08, 5.69), *p* < 0.0001, SUCRA = 75.7] showed relative superiority over RR and NR. The effect of PES [SMD = −0.35, 95% CI (−1.78, 1.07), *p* > 0.05, SUCRA = 7.7] was significantly poorer. This result suggests that although certain non-invasive stimuli may have some theoretical potential, in clinical practice they are not applied significantly beyond conventional treatment. In comparison to other studies, the results were different. tDCS was found to have a significant effect (*p* < 0.05) in improving QoL in a study by Palm et al. (2016), which may be related to the sample size and the duration of the intervention. The study by Bengisu et al. (2024), on the other hand, also reported the effectiveness of NmeS in improving the QoL of patients, which contrasts with our findings in the present study. This shows that the effect of non-invasive stimulation on QoL may be influenced by a variety of factors, including study design, sample characteristics, and mode of intervention.

### 4.3 Limitations

Our study acknowledges several limitations. Firstly, some comparisons were based on a limited number of studies, with certain interventions being supported by a sparse literature base. For instance, the NmeS group was underpinned by only three publications. Consequently, caution is warranted when interpreting these findings. Secondly, the temporal and geographical spread of the included literature inevitably introduced a degree of heterogeneity. Through analysis and discussion, we identified the underlying factors contributing to high heterogeneity and successfully mitigated it through subgroup analysis to a reasonable extent. While high heterogeneity could potentially affect the reliability and generalizability of the results, our refined subgroup analysis effectively addressed this concern. We anticipate that future research designs will achieve greater consistency, particularly in the selection of intervention frequency and duration, to minimize the impact of heterogeneity. Thirdly, due to the varying experimental designs and objectives across different interventions, the choice of scales for the primary outcome measure of swallowing function was not uniform. We assessed the risk of bias and data quality of these studies and determined that their impact on the overall results was limited. Lastly, network meta-analysis faces technical and theoretical limitations, such as the need for more sophisticated statistical methods and the challenge of resolving inconsistencies.

## 5 Conclusion

The results showed that rTMS was effective in improving swallowing function, and NmeS was the most effective in improving the QoL of stroke patients. These results suggest that rTMS and NmeS have significant advantages in improving swallowing function and QoL in stroke patients, respectively, pointing out the potential value of these two interventions in clinical practice and suggesting that they should be used as preferred intervention options in clinical practice. The combination of different interventions should be individualized based on the patient’s specific dysfunction and physical condition to promote functional recovery. Future studies should further explore the long-term effects of these interventions and search for optimal parameter configurations.

## Data Availability

The original contributions presented in the study are included in the article/supplementary material, further inquiries can be directed to the corresponding author.

## Glossary

RTD: Routine treatment of dysphagia
FDS: Functional Dysphagia Scale
RSR: Routine swallowing rehabilitation
FoiS: Functional Oral Intake Scale
BT: Behavioral therapy
VFSS: Video fluoroscopic swallowing study
ST: Swallowing training
DSRS: Dysphagia Severity Rating Scale
BR: Basic rehabilitation
PAS: Penetration Aspiration Scale
SC: Standard care
DOSS: Dysphagia Outcome and Severity Scale
NmeS: Neuro-muscular electrical stimulation
BI: Barthel index
PES: Pharyngeal electrical stimulation
SWAL-QOL: Quality of Life Assessment the Swallowing Quality of Life
rTMS: Repetitive transcranial magnetic stimulation
ASHA NOMS: American Speech-Language-Hearing Association National Outcome Measurement System
tDCS: Transcranial direct current stimulation
MASA: Modified Mann Assessment of Swallowing Ability
iTBS: Intermittent theta burst stimulation
